# Supplementary material for: Who belongs? Co-creating an assessment to measure belonging in a community space
Source: PLoS One. 2026 Apr 24;21(4):e0345864. doi: 10.1371/journal.pone.0345864 (PMC13108759; doi:10.1371/journal.pone.0345864)
Supplement: S1 Fig — (DOCX) [file pone.0345864.s001.docx]

**Figure 1**: **Semi-structured interview guide.** This guide was used in all focus groups to assess YMCA member conceptualization of belonging.

For this hour, I am going to ask you about your thoughts, feelings, and interpretations of the concept of belonging.

1. When you think about the word “belonging,” what comes to your mind?
2. How would you define belonging?
3. There are many definitions that exist for belonging. Each definition might or might not represent how you feel or think about it. One definition I found is: *a sense of fitting in or feeling like you are an important member of a group*
   1. When you hear this definition, what do you think about it? How does it make you feel? Is there something missing from this definition? If so, what?
4. How do you know when you belong?

Thank you for those responses. For the next questions, I’m going to shift gears a little bit.

1. What can help someone feel like they belong more?
2. What leads to someone feeling like they belong less?

For the next set of questions, I want you to think about a community you belong to.

1. How does your feeling of belonging to that community impact you?
2. How does your feeling of belonging to that community benefit that community?
3. What is the impact of a greater sense of belonging within a community as a whole?
