# Supplementary material for: Who belongs? Co-creating an assessment to measure belonging in a community space
Source: PLoS One. 2026 Apr 24;21(4):e0345864. doi: 10.1371/journal.pone.0345864 (PMC13108759; doi:10.1371/journal.pone.0345864)
Supplement: S1 Table — Column 1 is the demographic. Column 2 is the percent breakdown at the YMCA branch across all members. Column 3 is the percent breakdown of focus group participants. (DOCX) [file pone.0345864.s002.docx]

**Table 1: Belonging focus group participant demographics as compared to YMCA branch demographic makeup.** Column 1 is the demographic. Column 2 is the percent breakdown at the YMCA branch across all members. Column 3 is the percent breakdown of focus group participants.

|  | **YMCA Branch** | **Focus Group** |
| --- | --- | --- |
| **Age** |  |  |
| </=20 | 36% | 43% |
| 21-30 | 7% | 7% |
| 31-40 | 12% | 13% |
| 41-50 | 9% | 7% |
| >/=51 | 36% | 30% |
| **Race** |  |  |
| White | 64% | 43% |
| Black/AA | 28% | 50% |
| Native American | 0% | 0% |
| Asian | 0% | 7% |
| Pacific Islander | 0% | 0% |
| Other | 8% | 0% |
| **Gender** |  |  |
| Woman | 45% | 43% |
| Man | 55% | 53% |
| Trans-sexual | 0% | 0% |
| Non-binary | 0% | 0% |
| Other | 0% | 3% |
