# Supplementary material for: Who belongs? Co-creating an assessment to measure belonging in a community space
Source: PLoS One. 2026 Apr 24;21(4):e0345864. doi: 10.1371/journal.pone.0345864 (PMC13108759; doi:10.1371/journal.pone.0345864)

**Table 5**: **Mapping of validated surveys and focus group themes to final YMCA questionnaire.** Column 1 shows the phrasing of the question in the YMCA survey. Column 2 demonstrates the scale that we obtained the phrasing from. Column 3 demonstrates the qualitative theme that informed the question.


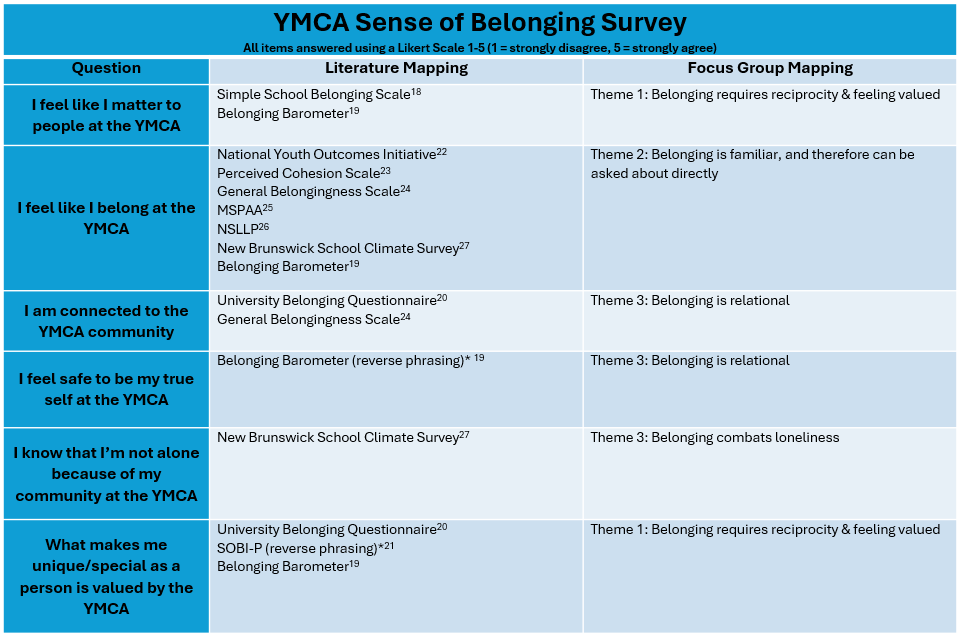

Supplement: S2 Table — Column 1 shows the phrasing of the question in the YMCA survey. Column 2 demonstrates the scale that we obtained the phrasing from. Column 3 demonstrates the qualitative theme that informed the question. (DOCX) [file pone.0345864.s003.docx]
