# Supplementary material for: Who belongs? Co-creating an assessment to measure belonging in a community space
Source: PLoS One. 2026 Apr 24;21(4):e0345864. doi: 10.1371/journal.pone.0345864 (PMC13108759; doi:10.1371/journal.pone.0345864)
Supplement: S3 Table — (DOCX) [file pone.0345864.s004.docx]

**Table 6: Quote tables.** Full quote tables for each theme, with associated codes and quotes.

| **Code** | **Source** | **Quote** |
| --- | --- | --- |
| **Theme 1: Belonging requires reciprocity** | | |
| In terms of effort and active engagement | *FG2, Speaker 6*  *FG3, Speaker 4* | *When they like. They invite you to like, do things. Like let’s say they have their original group, right? And you kind of like outside of their group. When someone like brings you in and invites you, brings you into the group, that makes you seem like you’re wanted*  *[Belonging] has to be cultivated. I don’t think it comes naturally. […] it’s work.* |
| In terms of value (feeling valued, contributing value) | *FG3, Speaker 3*  *FG4, Speaker 2* | *Feeling important can create a greater sense of belonging. Where I feel like I have been wanted, I feel more heavily drawn to.*  *I wouldn’t go so far as to say [needing to be] important, but I would say [having] something to contribute* |
| **Theme 2: The drive to belong is an innate part of being human** | | |
| Belonging is unanimously positive | *FG2, Speaker 6*  *FG5, Speaker 3*  *FG3, Speaker 3* | *You know, when I’m with them, like I feel more happy, more energized. Just, you know, better.*  *[Just being welcomed] with open arms, like a very positive environment.*  *It's going to be different for everyone in each place of belonging, but I would say like that general feeling of joy and comfort in those areas* |
| There is an immutable to drive to belong that exists in all humans | *FG5, Speaker 5*  *FG1, Speaker 5*  *FG1, Speaker 3* | *I mean, I think we're innately social beings that want to be, you know around other people, feel comfortable with other people.*  *We are social animals*  *God did not desire us to be alone* |
| **Theme 3: Belonging is relational and combats loneliness** | | |
| Connection & community are synonymous with belonging | *FG5, Speaker 4*  *FG5, Speaker 4*  *FG3, Speaker 3* | *I feel like if you have like a lack of like communicating with others that you don't feel like you're connected with them and that can make you feel like left out and stuff.*  *[Belonging is] being with people*  *Interaction with other people definitely drives community drives that belonging sense* |
| Belonging is embedded in trusting relationships, allowing one to be authentic | *FG2, Speaker 2*  *FG3, Speaker 4*  *FG3, Speaker 2* | *When you’re able to be yourself. When they like you for you*  *When I think about belonging. I would define it as like a safe space. Where you can just kind of come be yourself or you know if there's something that you just kind of need to get off your chest you know you have people that you can talk to in that space*  *I think sometimes if you don't feel like you belong, you can't be your true self. I also think it depends on what situation you're in, but um like being able to let your guard down. I think if you don't feel like you belong, it's hard to kind of let that guard down sometimes and show your true colors.* |
| Belonging is an antonym for loneliness | *FG2, Speaker 4*  *FG1, Speaker 5*  *FG1, Speaker 3* | *I feel like they can't help it for. Cuz they feel alone and they just find people to talk to and [try to belong]*  *It's a way to learn. To find out you're not alone.*  *You're not isolated - You belong. It's a part of you. You can reach out to somebody.* |
| **Theme 4: Sense of belonging is initiated and fostered through commonalities** | | |
| Shared adversity or pasts | *FG1, Speaker 5*  *FG1, Speaker 2* | *We had to hang in there for some really difficult transitions and it was a phenomenal experience. […] So we got to watch all of us grow up individually. We got to watch families grow up, we got to watch the hard parts that people went through and that's kind of. Rare.*  *Well, sharing common effects, you know, the common stories and the lives that you have with other people.* |
| Shared interests | *FG1, Speaker 8*  *FG3, Speaker 3* | *And they come from all different spectrums. But, you know, when you have some common interests, you kind of... You know, some of those divides kind of… the lines are blurred*  *I think that no matter how different everybody is, there is some common ground somewhere.* |
| Shared goals or purpose | *FG5, Speaker 5*  *FG3, Speaker 3* | *If you all have sort of the same purpose in mind um then then you all kind of work together to push for that.*  *I have a community with the members and the staff […] I find purpose in the swim lessons themselves, which always brings me back to the Y. Big reason why I've stayed here for eight years. I have another job that I get more money from, but this is where I find a lot more joy in my life of what I do* |
| **Theme 5: The benefits of belonging are many and multifaceted** | | |
| Individual benefit includes improved self confidence | *FG3, Speaker 5* | *I think it's good for you on like a lot of levels. Like if you walk away from a situation where you're like man, like those people really love me or I really love them. It’s good for your self esteem, it's good for your soul. You know, it's just all around good* |
| Individual benefit includes self growth and learning from others | *FG2, Speaker 2*  *FG1, Speaker 7* | *Like how can I explain it. You both belong, you both improve*  *You know, so I'm learning from them and they're learning from me.* |
| Communities benefit because members are more likely to give back | *FG3, Speaker 3*  *FG5, Speaker 3* | *I feel like you give more to places once you belong there. You're getting out of it more and you give that much more back.*  *Because I felt belonged, I felt welcomed, and now I could branch that welcoming sense to somebody else.* |
| Diversity of membership in a group creates a better and more cohesive whole | *FG2, Speaker 2*  *FG5, Speaker 5* | *Cause a community is supposed to be like different people and not everybody supposed to be the same, so they probably want to meet a new person*  *I mean it's the sum of the parts. So the more parts, the more good parts you have, the better it gets. […] I mean as long as people are bringing that sort of good energy. […] the more the merrier I guess.* |
| Communities with high belonging have increased recruitment and retention | *FG3, Speaker 3*  *FG5, Speaker 3* | *And it grows the community too. […] everybody kept talking, ‘You need to come here. You need to come here. You'll love this person. You can do this.’ And once we came - now that's what I'm saying to other people. ‘We love it here. You need to come here.’ […] we're excited to come here and so it gets that word out and that sense of belonging and that community is growing and getting bigger*  *I think in the less belonging, it's an in and out like the door is always, you know, revolving - it's in and out. Versus the more belonging environments, people are hanging around a lot longer. You know there's more coming in and less coming out.* |
| **Theme 6: Belonging is akin to membership** | | |
| Associating the feeling of belonging with being a part of a group | *FG3, Speaker 5*  *FG5, Speaker 3* | *They all, like, knew when my birthday was, you know, and got me something. That's something that I can point to as like an aha moment where I think I really felt like I was part of like part of that group*  *Like I took my son to the workout class downstairs last week. And even like the instructor was going around like fist bumping. You know everybody. And he's 12, and these, you know, other adults 30-40 fifty year olds in there and he's hanging with him and all the adults were like, man, he was smiling the whole time. He was so excited. […] And there's just that positive feedback. You just feel good no matter what* |
| Associating welcoming spaces with spaces of high belonging | *FG5, Speaker 5*  *FG3, Speaker 4* | *I think knowing a name or a face and being comfortable with just like walking up to them […] Even though I don't know your name or anything about you besides you take this class but I feel comfortable enough to, you know, strike up a quick conversation before it starts or you know, obviously it's better if you know someone's name. It makes it easier. But even just a face a friendly face*  *I think just a welcoming environment. […] I feel like if there's someone that's just like, ‘hey, how are you doing’ or just have fun and just take the time to speak directly to you instead of saying hi and you just keep on going. I think that works, that helps […] And just being welcomed because I can come in somewhere and nobody say anything and just sit in my corner and you know, go and do stuff and it doesn't seem like a sense of belonging.* |
