# Supplementary material for: Who belongs? Co-creating an assessment to measure belonging in a community space
Source: PLoS One. 2026 Apr 24;21(4):e0345864. doi: 10.1371/journal.pone.0345864 (PMC13108759; doi:10.1371/journal.pone.0345864)
Supplement: Attachment — Submitted filename: Community engagement.docx [file pone.0345864.s005.docx]

Community engagement

Add citation to your method

Move the lit review to before the method

You are not doing a systematic or scoping review—eliminate where you found your references—the reader assumes you did you work in finding them---this is a different type of lit review

However, you need to develop and expand on a lit review rather than simply a discussion of what you looked for

Given, that you are creating an assessment you need to talk about how you did that

What items?, how did you pilot them? What is the validity and reliability?

You must bring the lit review and survey development OUT of the supplemental materials and into the paper

You need much more detail about HOW you did your thematic analysis—I recommend Braun and Clarke

Your quotes also have to be integrated into the paper and not in the supplemental section

Y ou have some examples, but after you add those direct quotes the paragraph needs a concluding sentence at minimum

Matching the items to prior assessment is part of construct validity—you need to present that in different ways
