# Supplementary material for: Who belongs? Co-creating an assessment to measure belonging in a community space
Source: PLoS One. 2026 Apr 24;21(4):e0345864. doi: 10.1371/journal.pone.0345864 (PMC13108759; doi:10.1371/journal.pone.0345864)
Supplement: Attachment — Submitted filename: Belonging paper reviewer response_submit.docx [file pone.0345864.s007.docx]

PLOS One Reviewer comments & responses

| **Formatting & cover page:** | |
| --- | --- |
| Please ensure that your manuscript meets PLOS ONE's style requirements, including those for file naming. The PLOS ONE style templates can be found at | Thank you for bringing our attention to this. We have worked to adjust the paper style to fit PLOS One formatting requirements. |
| Please note that funding information should not appear in any section or other areas of your manuscript. We will only publish funding information present in the Funding Statement section of the online submission form. Please remove any funding-related text from the manuscript. | We appreciate the clarification and have removed any funding-related text from the manuscript. |
| Please expand the acronym “NIH” (as indicated in your financial disclosure) so that it states the name of your funders in full.  This information should be included in your cover letter; we will change the online submission form on your behalf. | We have expanded the acronym for our cover letter, and removed funding information from the manuscript. |
| We note that your Data Availability Statement is currently as follows: “All relevant data are within the manuscript and its Supporting Information files.”  Please confirm at this time whether or not your submission contains all raw data required to replicate the results of your study. Authors must share the “minimal data set” for their submission. PLOS defines the minimal data set to consist of the data required to replicate all study findings reported in the article, as well as related metadata and methods ([https://journals.plos.org/plosone/s/data-availability#loc-minimal-data-set-definition [journals.plos.org]](https://urldefense.com/v3/__https:/journals.plos.org/plosone/s/data-availability*loc-minimal-data-set-definition__;Iw!!FMDslA!b0Mww7su2ir-uL7daetCWHxw6QvWDcjtu_iIvw4-7K-_mq3_mIA3cqr9ky2_aeD608KE0_GqmPSn4OBQCbLjQ10eNrIumU8$)).  If there are ethical or legal restrictions on sharing a de-identified data set, please explain them in detail.  We note that this data set consists of interview transcripts. Can you please confirm that all participants gave consent for interview transcript to be published? | We understand and appreciate the need for transparency. PLOS recommendations for qualitative data sharing include:  “For studies analyzing data collected as part of qualitative research, authors should make excerpts of the transcripts relevant to the study available in an appropriate data repository, within the paper, or upon request if they cannot be shared publicly. If even sharing excerpts would violate the agreement to which the participants consented, authors should explain this restriction and what data they are able to share in their Data Availability Statement.”  We additionally reviewed standards for reporting and data sharing of qualitative work per three highly-cited sources:   - Yarris LM, Balmer D, Gottlieb-Smith R, Sullivan GM. Editors' Guidance for Submitting Qualitative Research to the Journal of Graduate Medical Education. J Grad Med Educ. 2024 Jun;16(3):246-250. doi: 10.4300/JGME-D-24-00389.1. Epub 2024 Jun 13. PMID: 38882410; PMCID: PMC11173034. - O’Brien, Bridget C. PhD; Harris, Ilene B. PhD; Beckman, Thomas J. MD; Reed, Darcy A. MD, MPH; Cook, David A. MD, MHPE. Standards for Reporting Qualitative Research: A Synthesis of Recommendations. Academic Medicine 89(9):p 1245-1251, September 2014. \| DOI: 10.1097/ACM.0000000000000388 - Tong, A., Sainsbury, P., & Craig, J. (2007). Consolidated criteria for reporting qualitative research (COREQ): a 32-item checklist for interviews and focus groups. International Journal for Quality in Health Care, 19(6), 349-357.   In alignment with all of the above sources and PLOS recommendations, we confirm at this time that excerpts of the transcripts relevant to the study, the moderator guide, and our analysis are available within the manuscript and supplementary materials. We believe that this level of data availability is sufficient for readers to replicate our study if so desired, highlighting that their community of interest might define different themes of belonging than our community and therefore it is the method of analysis rather than the raw transcript themselves that would provide replicability.  In addition to it not being standard to provide raw transcripts within qualitative data publishing, we will abstain from providing raw transcripts for two further reasons: we did not ask participants for consent for this, and the small sample size of participants and local context (one branch of the Cincinnati YMCA) creates a situation in which even de-identified raw transcripts of individuals thoughts and feelings can then be easily identified. |
| Your ethics statement should only appear in the Methods section of your manuscript. If your ethics statement is written in any section besides the Methods, please move it to the Methods section and delete it from any other section. Please ensure that your ethics statement is included in your manuscript, as the ethics statement entered into the online submission form will not be published alongside your manuscript. | Thank you for the feedback, we have incorporated this information into the methods section and removed it from the cover page. |
| Please include captions for your Supporting Information files at the end of your manuscript, and update any in-text citations to match accordingly. Please see our Supporting Information guidelines for more information: [http://journals.plos.org/plosone/s/supporting-information [track.editorialmanager.com]](https://urldefense.com/v3/__http:/track.editorialmanager.com/CL0/http:*2F*2Fjournals.plos.org*2Fplosone*2Fs*2Fsupporting-information/1/010f0199ff945209-f56d1534-d1b5-4f30-8a09-5b4c7c927a99-000000/TfPfKbXDs9LvV7rq3FTWxKsqMn73et-7BfuuGvuPM2Q=233__;JSUlJSU!!FMDslA!b0Mww7su2ir-uL7daetCWHxw6QvWDcjtu_iIvw4-7K-_mq3_mIA3cqr9ky2_aeD608KE0_GqmPSn4OBQCbLjQ10ei87uA2Y$). | Thank you for calling attention to this. We have adjusted our incorporation of tables and figures to fit PLOS guidelines. |
| Please remove your figures from within your manuscript file, leaving only the individual TIFF/EPS image files, uploaded separately. These will be automatically included in the reviewers’ PDF | Thank you for this recommendation, we have updated our manuscript accordingly. |
| If the reviewer comments include a recommendation to cite specific previously published works, please review and evaluate these publications to determine whether they are relevant and should be cited. There is no requirement to cite these works unless the editor has indicated otherwise. | Reviewer recommended adjusting methods section to align with Braun and Clarke. We found that this was a relevant and fitting model to report our qualitative analytic method and have adjusted our methods report to fit the model accordingly and with appropriate citation. |
| **Overall paper structure** | |
| It appears that you are doing two different things: one is a qualitative study of people's view of belonging while the other is the development of an assessment. I strongly recommend removing the assessment piece as this time as there is much more work to do to get reliability and validity of an assessment. | This suggestion was reviewed with the editor via email. We have included qualitative work and assessment creation with better introduction to the goal (participatory action research, community informed survey creation) and method description (statistical analysis showing survey validity, qualitative analysis description) |
| The authors use "wellbeing" and "well-being" in different part of the manuscript; would be consistent in using one or the other | Thank you for pointing this discrepancy out, we have updated the manuscript to be uniform in our use of “wellbeing”. |
| **Introduction** | |
| I would encourage the authors to push further on the need for more broad survey tool development that better reflects actual community perspectives; particularly if belonging is to be measured as a tool to drive interventions that can improve health. | We appreciate this insight and have included further description on the importance of CBPR approaches to research (including the feedback from community’s that research timelines often don’t fit community change timelines) into the introduction. We have additionally provided further background on the lack of community voice in survey development of belonging to-date, thus highlighting an opportunity for work such as ours to be elevated. |
| Would consider including a citation for CBPR as this is a model paper it can direct readers to learn about this approach and ensure they are true to the method when engaging in this work in their communities; particularly as this will reach an academic audience | We appreciate this recommendation, and have provided additional information about CBPR in our introduction including citations for the reader to have a better understanding. |
| **Methods** | |
| Move the lit review to before the method | We appreciate this recommendation and have made the necessary changes. |
| For the Literature Review, was there a timeframe for the search or any articles from any year past were included? | Timeframe of article publication was not a factor in our review of the literature. Language is now included in the manuscript to note this. |
| You must bring the lit review and survey development OUT of the supplemental materials and into the paper | We appreciate this feedback and have adjusted the manuscript to ensure that there is no new information in the supplemental materials that have not been fully described and presented in the manuscript within each discussed theme. |
| Your quotes also have to be integrated into the paper and not in the supplemental section | Thank you for this recommendation. We have provided supporting quotes for each theme within the paragraphs of the manuscript. Our full quote tables found in the supplementary materials provides supplemental quotes that additionally informed the theme without overwhelming the manuscript within text. |
| The lit review, the method, the analysis all need much more detail--some suggestions in the attached file.   - I recommend you access other qualitative articles and see how thematic analysis are conducted. Your results are close but the how you got there needs expanded. You need to expand your own positionally as well. | We are grateful for this feedback and have significantly adjusted the manuscript to provide more detail and background on the outlined sections. This includes incorporation of the Braun & Clarke steps of thematic analysis, further quantitative validation of our survey, and ensuring that our positionality is well described. |
| You need much more detail about HOW you did your thematic analysis—I recommend Braun and Clarke | We agree that the Braun & Clarke steps of thematic analysis fit our methods well and have updated our methods section to fit their steps. |
| If you want to focus on the development of the assessment--please look at how other measures/surveys are developed. | This recommendation was discussed with the editor via email. We sought to expedite novel survey creation as one of the contributions of our paper and thus did not pursue full validation of the survey items per standard assessment development. That said, we recognize that our methods and results were lacking confirmation that our method was successful and we have therefore included a Cronbach alpha analysis of our survey to assess validity. This additional information has been added to the manuscript |
| You are not doing a systematic or scoping review—eliminate where you found your references—the reader assumes you did you work in finding them---this is a different type of lit review | We appreciate this feedback and have incorporated suggested edits. |
| You need to develop and expand on a lit review rather than simply a discussion of what you looked for | We appreciate the encouragement. We have expanded our description of the literature review as recommended. |
| Add citation to your method | We appreciate this recommendation and have included citations for our methodology within the manuscript. |
| Line 186 (start of qualitative analysis section) --> given focus on local community context, would further specify location of this YMCA such as by rewording to "This work is a case study examining how Cincinnati YMCA members experience.." or "...how members of a local YMCA experience..." | We appreciate this feedback and have eliminated the sentence for other flow-related reasons. |
| Lines 202/203 --> please clarify if it was important for project team members to understand or agree/come to consensus on the themes" | This paragraph is restructured in aligning qualitative method reporting with current norms. No longer relevant. |
| Lines 243-245 --> "external member checking" is not fully clear in terms of what was done to validate the survey; was it given to members of nearby YMCA branches to complete? did these branches reflect the same community demographics? Why was the same YMCA membership not used through individuals not in the focus group if the goal is in-community construction and utilization? If the nearby branches are considered part of the same community might be of benefit to explain this further. As it reads, not fully clear what measurement validity included. | We agree that our methods were not fully described and have provided more detail of our analytic methods to give further clarification. |
| Is the community project partner being trained by the study lead a possible limitation of the study given these were the 2 who then did the initial theme evaluation? Not sure what is standard for qualitative projects to this may not have any bearing. | We appreciate the question, and have not considered this training approach to be a limitation of the study. |
| **Results** | |
| Additionally, the 3 themes that did not map to validated survey structures seem to be the meat of the authors findings but are not really explored or further measured by the ultimate survey that was created. If individuals were to mimic this study, the authors should give guidance for what groups should do if similarly multiple themes on belonging to not map to current validated studies. | Thank you for this feedback. We have updated our description of the themes, as well as our discussion, to further describe why the three mentioned themes were not part of the developed survey. The reasons for each exclusion were different, and we hope that our updated description will help other individuals/researchers in identifying reasons to include/exclude themes within their own surveys. |
| You have some examples, but after you add those direct quotes the paragraph needs a concluding sentence at minimum | We appreciate the encouragement to ensure adequate contextualization of direct quotations. In some instances, we have added language after the quotation. In other instances, however, we included all contextualization prior to the quotation and are concerned that further language after the quotation would read as redundant. |
| What items?, how did you pilot them? What is the validity and reliability? | We updated our methods to further describe how our survey was piloted, and additionally provided quantitative analytics (Cronbach alpha) that suggests good internal consistency of the items. |
| Matching the items to prior assessment is part of construct validity—you need to present that in different ways | Thank you for this recommendation. We hope that the updates to our methods and results better describes how we approached construct validity. |
| Lines 259-260 --> Important information in results so would remove the parenthesis and just state these results. | Thank you for this edit suggestion, we have incorporated it into our manuscript. |
| Line 261-262 --> suggested rewording here: "Thematic analysis revealed six major themes about belonging (see Supplemental Materials for quote tables)." | Thank you for this edit suggestion, we have incorporated it into our manuscript. |
| Table 2, 3, and 4 --> slightly unclear if the Survey Item Phrasing are examples from the existent scales or ones that the authors created for their own survey; consider re-naming column "Existent Survey Item Phrasing" to make clear to the reader | Thank you for this edit suggestion, we have incorporated it into our manuscript. |
| Line 305-307 and Table 3 --> Don't fully follow the logic from belonging being positive/well known to it therefore being asked directly. Can the authors draw this connection out further? (quite different from Theme 1/table 2 and Theme 3/Table 4. | Thank you for this edit suggestion, we have reworded the paragraph to provide better flow of our assertion that a well known construct can be asked about directly. |
| Line 324 --> "This ___ is fostered through..." -- can the authors clarify what "This" is referring to? maybe rewording as "This high belong is fostered..." or "This ability to be truthful is fostered..." | Thank you for this edit suggestion, we have taken out “this” and better described what we’re talking about in the sentence mentioned. |
| Line 348 and 350 --> "This was true..." and "This was similarly seen..." -- can the authors clarify what "This" is referring to in each segment; given multiple statements prior best to be specific as to what the quotations are supporting. consider: "This commonality as a drive of belonging was true for recreational..." and "This expression of shared feelings was similarly seen..." | Thank you for this edit suggestion, we have taken out “this” and better described what we’re talking about in the sentence mentioned. |
| Lines 368-370 --> why didn't this information inform the measurement of sense of belonging? Can the authors explain this further? Or why was it necessary for the research team if not part of the measured objective? | Thank you for these clarifying questions. We have provided further detail in the sections mentioned to explain why a theme of the qualitative data analysis did not get incorporated into the survey. |
| Theme 5 and 6 --> would include something similar to Theme 4 that this theme was not reflected in existent surveys or why it was otherwise not used to generate the community-specific belonging survey by the authors | We agree that this was not clear and have provided further explanation as to the reasons why we did not incorporate these themes into survey development. |
| Line 398 --" again use of "This" without clarity on what it is referencing; suggestion "This connection between belonging and group member was seen when participants..." | Thank you for this edit suggestion, we have taken out “this” and better described what we’re talking about in the sentence mentioned. |
| **Discussion** | |
| Line 442 --> Not sure if the focused-on themes were nuanced and evolving as they were reflected in existing validated tools? Maybe the approach to create a focused survey reflects the specific community perspectives and values. The 3 themes not reflected are very interesting in terms of "evolving" ways that belonging is described and viewed. | We appreciate this suggestion and have edited the sentence to provide more clarity. |
| Line 447 & 454 & 467 --> again would clarify what "This" in the sentence "This included belonging..." is referring to and what "This was seen by..." is referring to and what "This could be..." is referring to | Thank you for this edit suggestion, we have taken out “this” and better described what we’re talking about in the sentence mentioned. |
| line 452 --> keep study description/findings in past tense for consistency; "additional observation we make" to "we made" | We have updated our manuscript to ensure consistency in tense. |
| *not including themes 4-6 as a study limitation?? leaving out whole aspects of this community's definition or expression of belonging | We agree that this was unclear in our original manuscript draft and have provided further information on why a theme that stemmed from our qualitative analysis did not get incorporated into the survey. We have additionally discussed this as a limitation of the study. |
