# Supplementary material for: Who belongs? Co-creating an assessment to measure belonging in a community space
Source: PLoS One. 2026 Apr 24;21(4):e0345864. doi: 10.1371/journal.pone.0345864 (PMC13108759; doi:10.1371/journal.pone.0345864)
Supplement: Attachment — Submitted filename: Belonging paper reviewer response_030826.docx [file pone.0345864.s008.docx]

PLOS One Reviewer comments & responses

| **Overall paper structure, diction, syntax** | |
| --- | --- |
| We note that your Data Availability Statement is currently as follows: “All relevant data are within the manuscript and its Supporting Information files.”  Please confirm at this time whether or not your submission contains all raw data required to replicate the results of your study. Authors must share the “minimal data set” for their submission. PLOS defines the minimal data set to consist of the data required to replicate all study findings reported in the article, as well as related metadata and methods ([https://journals.plos.org/plosone/s/data-availability#loc-minimal-data-set-definition [journals.plos.org]](https://urldefense.com/v3/__https:/journals.plos.org/plosone/s/data-availability*loc-minimal-data-set-definition__;Iw!!FMDslA!b0Mww7su2ir-uL7daetCWHxw6QvWDcjtu_iIvw4-7K-_mq3_mIA3cqr9ky2_aeD608KE0_GqmPSn4OBQCbLjQ10eNrIumU8$)).  For example, authors should submit the following data: - The values behind the means, standard deviations and other measures reported; - The values used to build graphs; - The points extracted from images for analysis.  Authors do not need to submit their entire data set if only a portion of the data was used in the reported study.  If there are ethical or legal restrictions on sharing a de-identified data set, please explain them in detail. | We understand and appreciate the need for transparency. We have previously addressed data availability statement questions as it relates to our qualitative data (please let us know if that needs to be further outlined or resent), and believe this new comment is in regard to the Cronbach alpha analysis that was performed to assess for internal validity.  The data used to perform the Cronbach alpha are owned by a third-party organization (the YMCA of Greater Cincinnati) and is a small part of a larger survey instrument. We would welcome requests for values within the dataset, and the YMCA of Greater Cincinnati can consider requests on a case-by-case basis. These requests can be sent to the corresponding author on this paper ([carley.riley@cchmc.org](mailto:carley.riley@cchmc.org)) who would be responsible for bringing the request to the YMCA of Greater Cincinnati team. |
|  | Thank you for this feedback. We eliminated hyphens where able, though continued to use them when the two-word combinations were used as an adjective. |
| No contractions outside of quotes | We have reviewed the manuscript and removed contractions that existed outside of direct focus group quotes. |
| Use the COREQ checklist which is on the Plos One website to highlight the following six issues   1. Defined objective/research question 2. Description of sampling strategy, rational for recruitment, inclusion & exclusion procedures, and number of participants 3. Data collection procedures 4. Data analyses procedure to enable replication 5. Potential bias 6. Limitations | We appreciate the need for rigor in evaluating qualitative work. We report on our compliance with the 6 identified COREQ checklist items below, specifically focusing on our reporting of the qualitative portion of our work:   1. We did not clearly describe our objective for the qualitative work. This has been revised, with new language under the subheading of “Focus Group”, lines 155-158 2. Our sampling strategy and rational can be found under the subheading of “Focus Groups”, lines 158-174 3. Our data collection procedures can be found under the subheading of “Focus Groups”, lines 178-192 4. The approach we used for data analyses can be found within the “Qualitative Analysis” subheading, lines 195-214 5. Potential bias was considered and addressed through the process of research reflexivity. Description of this process can be found under the subheading “Research Reflexivity” lines 217-240 6. The limitations of our study, particularly as it relates to our qualitative sampling, can be found within the “Discussion” section, particularly lines 543-554 |
| **Abstract & Introduction** | |
| A sense of belonging – need an article | We have included a citation for where we obtained this definition. |
| Wellbeing is well-established (need citations when you say this) | Thank you for providing opportunity for clarification. We believe you are referencing the text in line 76. We provide the evidence for the links between belonging and wellbeing in the subsequent sentences (lines 77-83). |
| Line 63 – take out “and” between literature review and extraction | Thank you for this edit, it has been incorporated in the revised manuscript. |
| **Methods** | |
| 191 is not literature review – take out that heading | Line 191 from the initial revision is the first line of text below the subheading Qualitative Analysis. We suspect that the reviewer is talking about the subheading “Literature Review”, which we have renamed to “Existing Literature”. If we misinterpreted the feedback, please let us know. |
| **Results** | |
| Line 380 not sure what is happening with that table and then going into the next theme – same as Table 3 | Line 380 is text within Theme 4, and does not include reference to a table. We deduced that the reviewer is remarking upon the embedded Tables 2-4 higher up within the manuscript, but it is not clear from the feedback what is felt to be missing. Under the thought process that it is potentially related to unclear labels for each table, we have revised the Table description for Tables 2, 3, and 4. If alternative feedback was meant, we welcome correspondence regarding this and will make adjustments as necessary. |
| Concluding sentences after direct quotes to end a paragraph | We appreciate this feedback, and have included additional text within the revised manuscript to conclude paragraphs so that they do not end with direct quotes. |
| This is the finding of items for your measure – give it a better heading | We are more than happy to improve headings, but we are not certain to what heading this feedback refers. We are also happy to have the editor choose a better heading. |
